# Supplementary material for: Individual responses to a single oral dose of albendazole indicate reduced efficacy against soil-transmitted helminths in an area with high drug pressure
Source: PLoS Negl Trop Dis. 2021 Oct 19;15(10):e0009888. doi: 10.1371/journal.pntd.0009888 (PMC8555840; doi:10.1371/journal.pntd.0009888)
Supplement: S1 Text — (DOCX) [file pntd.0009888.s001.docx]

## S1 Text Details of the Bayesian statistical model used to estimate individual egg reduction rates

We defined a negative binomial mixed model motivated by the approach outlined in Crellen et al. [1] and Walker et al. [2, 3]. The model is defined as

| $y_{hij}\sim\text{ NB}\left( \boldsymbol{\mu}_{ij},\phi\right)$ |  |
| --- | --- |
| ${log(\boldsymbol{\mu}}_{ij})=\boldsymbol{\beta}\mathbf{X}_{i} \boldsymbol{+}\mathbf{b}_{i}\mathbf{Z}_{i}\boldsymbol{+}b_{j}$ |  |
| $\mathbf{b}_{i}\boldsymbol{\sim}\mathcal{N}\left( \boldsymbol{0},\boldsymbol{\Sigma} \right)$ |  |
| $b_{j}\boldsymbol{\sim}\mathcal{N}\left( 0,\sigma^{2} \right)$ | (1) |

Here, $y_{hij}$denotes a fecal egg count (FEC) $h$ measured from participant $i$ from school $j$ which is assumed to be a realization from a negative binomial distribution with mean $\boldsymbol{\mu}_{ij}$ and overdispersion parameter $\phi$. Parameter $\phi$ permits extra-Poisson variation among the duplicate FECs measured from the same individual at the same time point (before or after administration of albendazole). The mean $\boldsymbol{\mu}_{ij}=\left\{ \mu_{1ij},\mu_{2ij} \right\}$, comprising terms associated with FECs measured before (baseline) and after (follow up) treatment respectively, is modelled as a log-linear combination of fixed and random effects. The design matrix $\mathbf{X}_{i}$ comprises fixed effects terms with associated coefficients $\boldsymbol{\beta=}\left\{ \beta_{0},\beta_{1},\ldots,\beta_{m} \right\}$. Specifically, $\mathbf{X}_{i}$ includes indicator variables for time point (baseline or follow up), study site (Ethiopia, Lao PDR and Pemba Island), age (<9, 9-11, 12-15), sex, follow up time (> 2 weeks or ≤2 weeks), co-infection status and interactions between time point and the other variables. The log-linear structure means that coefficients associated with FECs measured after treatment quantify the multiplicative change in FECs compared to baseline. That is, the greater the coefficient, the *smaller* the egg reduction rate (ERR). The interactions between the fixed effects and the binary indicator variable of treatment quantify the effect of each fixed effect term on the treatment response (i.e. on the ERR). The random effects design matrix $\mathbf{Z}_{i}$ permits incorporation of an individual-specific intercept and ‘gradient’ $\mathbf{b}_{i}\boldsymbol{=}\left\{ b_{0i},b_{1i} \right\}$ associated with FECs measured before and after treatment respectively. These are assumed to follow a bivariate normal distribution with mean 0 and variance-covariance matrix $\boldsymbol{\Sigma}$. The diagonal components of $\boldsymbol{\Sigma}$ quantify, respectively, variance among individuals’ FECs (not explained by the fixed effects) and variance in their response to treatment. The off-diagonal components quantify covariance between the intercept and ‘gradient’ terms ($b_{0i}$ and $b_{1i}$). That is, correlation between individuals’ FECs measured at baseline and their subsequent response to treatment. Finally, $b_{j}$ is an additional random effects intercept term, modelled as normally distributed with mean 0 and variance $\sigma^{2}$, accounting for any clustering of FECs among individuals attending the same school.

We fitted the model to the FECs using Markov chain Monte Carlo sampling in Stan [4] using the brms package [5, 6] for R version 3.6.2 [7]. The model was fitted separately to the FEC data from each soil-transmitted helminth infection (*Ascaris lumbricoides, Trichuris trichiura* and hookworm). We defined vague normal priors for the fixed effects coefficients $\boldsymbol{\beta}$ with mean 0 and variance 10^6^ and default half student-t priors with 3 degrees of freedom for variance-covariance terms [6]. The overdispersion parameter of the negative binomial distribution was assigned a gamma prior with shape and rate parameters set to 0.01. We initialized three independent Markov chains, running each for 3,000 iterations and discarding the first 1,000 iterations as ‘burn in’. Hence, for each model we obtained 6,000 samples from the parameter posterior distribution. We assessed convergence of the chains using the $\hat{R}$ convergence diagnostic, confirming that $\hat{R}$ for each parameter was ≤ 1.05 [8-10] (see S2, S3 and S4 Tables).

## References

1. Crellen T, Walker M, Lamberton PH, Kabatereine NB, Tukahebwa EM, Cotton JA, et al. Reduced Efficacy of Praziquantel Against *Schistosoma mansoni* Is Associated With Multiple Rounds of Mass Drug Administration. Clin Infect Dis. 2016;63(9):1151-9. Epub 2016/07/30. doi: 10.1093/cid/ciw506. PubMed PMID: 27470241; PubMed Central PMCID: PMCPMC5064161.

2. Walker M, Churcher TS, Basáñez M-G. Models for measuring anthelmintic drug efficacy for parasitologists. Trends Parasitol. 2014;30(11):528-37. Epub 2014/09/15. doi: 10.1016/j.pt.2014.08.004. PubMed PMID: 25217844.

3. Walker M, Mabud TS, Olliaro PL, Coulibaly JT, King CH, Raso G, et al. New approaches to measuring anthelminthic drug efficacy: parasitological responses of childhood schistosome infections to treatment with praziquantel. Parasit Vectors. 2016;9:41. Epub 2016/01/28. doi: 10.1186/s13071-016-1312-0. PubMed PMID: 26813154; PubMed Central PMCID: PMCPMC4728951.

4. Stan Development Team. Stan Modeling Language Users Guide and Reference Manual, version 2.26. 2020.

5. Bürkner P-C. Advanced Bayesian multilevel modeling with the R: package brms. R J. 2018;10:395-411. doi: 10.32614/RJ-2018-017.

6. Bürkner P-C. brms: An R packages for Bayesian multilevel models using Stan. J Stat Softw. 2017;80:1-28. doi: 10.18637/jss.v080.i01.

7. R Core Team. R: A Language and Environment for Statistical Computing. Vienna, Austria: R Foundation for Statistical Computing; 2019.

8. Brooks SP, Gelman A. General methods for monitoring convergence of iterative simulations. J Comp Graph Stat. 1997;7:434-55.

9. Vehtari A, Gelman A, Simpson D, Carpenter B, Bürkner P-C. Rank-Normalization, Folding, and Localization: An Improved R-hat for Assessing Convergence of MCMC. Bayesian Anal. 2021;Advance Publication:1-28. doi: 10.1214/20-BA1221.

10. Gelman A, Rubin DB. Inference from iterative simulation using multiple sequences. Stat Sci. 1992;7:457-511.
