# Supplementary material for: Individual responses to a single oral dose of albendazole indicate reduced efficacy against soil-transmitted helminths in an area with high drug pressure
Source: PLoS Negl Trop Dis. 2021 Oct 19;15(10):e0009888. doi: 10.1371/journal.pntd.0009888 (PMC8555840; doi:10.1371/journal.pntd.0009888)
Supplement: S1 Table — (DOCX) [file pntd.0009888.s002.docx]

S1 Table. The estimated percentage of individuals with an a ‘satisfactory’ response. Percentages are derived from the posterior distribution of the individual egg reduction rate, denoted ${ERR}_{i}$, estimated using the Bayesian model fitted to fecal egg count data on *Ascaris lumbricoides,* *Trichuris trichiura* and hookworm collected before and after administration of a single 400 mg oral dose of albendazole in three study sites, Ethiopia, Lao PDR and Pemba Island (Tanzania). Individuals were treated (*N_+_*) if they were positive for any of the three infections by any of the four diagnostics (duplicate Kato-Katz thick smear, Mini-FLOTAC, FECPAKG2 and qPCR).

| Study site | *Ascaris lumbricoides* | |  | *Trichuris trichiura* | |  | hookworm | |
| --- | --- | --- | --- | --- | --- | --- | --- | --- |
|  | *N_+_* | Percentage ${ERR}_{i}\geq95\%$  (95% CrI^a^) |  | *N_+_* | Percentage ${ERR}_{i}\geq90\%$  (95% CrI) |  | *N_+_* | Percentage ${ERR}_{i}\geq80\%$  (95% CrI) |
| Ethiopia | 137 | 98.5  (97.1, 99.3) |  | 106 | 65.1  (58.5, 71.7) |  | 90 | 85.6  (78.9, 90.0) |
| Lao PDR | 111 | 96.4  (94.6, 96.4) |  | 105 | 61.0  (54.3, 67.6) |  | 228 | 82.0  (79.8, 84.6) |
| Pemba Island | 193 | 95.3  (92.7, 97.0) |  | 245 | 29.0  (26.1, 31.8) |  | 139 | 63.3  (56.1, 69.8) |

^a^ credible interval
