# Supplementary material for: Individual responses to a single oral dose of albendazole indicate reduced efficacy against soil-transmitted helminths in an area with high drug pressure
Source: PLoS Negl Trop Dis. 2021 Oct 19;15(10):e0009888. doi: 10.1371/journal.pntd.0009888 (PMC8555840; doi:10.1371/journal.pntd.0009888)
Supplement: S2 Table — (DOCX) [file pntd.0009888.s003.docx]

S2 Table. Coefficient estimates for the negative binomial mixed effects model fitted to *Ascaris lumbricoides* fecal egg counts. Estimates derived from the Bayesian model fitted to fecal egg count data on *Ascaris lumbricoides* collected before and after administration of a single 400 mg oral dose of albendazole in three study sites, Ethiopia, Lao PDR and Pemba Island (Tanzania).

| **Variable** | **Posterior mean (95% Crl^a^)** | $\hat{R}$ |
| --- | --- | --- |
| Intercept | 2.67 (0.71, 4.38) | 1.02 |
| Country |  |  |
| Ethiopia | 0^b^ | NA^c^ |
| Lao PDR | 0.90 (-0.89, 3.01) | 1.02 |
| Pemba Island | 0.24 (-1.62, 2.23) | 1.03 |
| Sex |  |  |
| Female | 0 | NA |
| Male | 0.42 (-0.34, 1.18) | 1.02 |
| Age |  |  |
| 6-9 years | 0 | NA |
| 10-12 years | -0.34 (-1.07, 0.33) | 1.01 |
| 13-14 years | -1.21 (-2.08, -0.33) | 1.02 |
| Coinfection |  |  |
| Single infection | 0 | NA |
| *Trichuris trichiura* | 0.95 (0.17, 1.78) | 1.05 |
| hookworm | 0.38 (-0.35, 1.03) | 1.05 |
| Treatment |  |  |
| Baseline | 0 | NA |
| Follow-up | -23.86 (-25.90, -15.63) | 1.01 |
| Country*Treatment |  |  |
| Ethiopia | 0 | NA |
| Lao PDR | 8.90 (2.80, 16.95) | 1.01 |
| Pemba Island | 5.56 (0.62, 11.51) | 1.00 |
| Sex*Treatment |  |  |
| Female | 0 | NA |
| Male | -5.33 (-10.25, -1.54) | 1.00 |
| Age*Treatment |  |  |
| 6-9 years | 0 | NA |
| 10-12 years | -2.86 (-7.17, 1.09) | 1.02 |
| 13-14 years | -10.13 (-20.93, -2.39) | 1.01 |
| Follow up*Treatment |  |  |
| > 2 weeks | 0 | NA |
| 1-2 weeks | 2.49 (-1.72, 7.00) | 1.01 |
| Coinfection*Treatment |  |  |
| Single infection | 0 | NA |
| *Trichuris trichiura* | 3.55 (-2.38, 11.05) | 1.01 |
| hookworm | -0.64 (-5.42, 3.82) | 1.00 |
| *Random effects hyperparameters* |  |  |
| SD^d^ individual intercept | 3.23 (2.98, 3.48) | 1.01 |
| SD individual treatment response | 8.92 (6.55, 12.59) | 1.00 |
| Correlation intercept & treatment response | -0.17 (-0.39, 0.10) | 1.00 |
| SD school intercept | 0.82 (0.04, 2.27) | 1.00 |
| Overdispersion parameter | 25.66 (21.36, 30.67) | 1.00 |

^a^ credible interval; ^b^ coefficient for reference category set to 0; ^c^ not applicable; ^d^ standard deviation
