# Supplementary material for: Individual responses to a single oral dose of albendazole indicate reduced efficacy against soil-transmitted helminths in an area with high drug pressure
Source: PLoS Negl Trop Dis. 2021 Oct 19;15(10):e0009888. doi: 10.1371/journal.pntd.0009888 (PMC8555840; doi:10.1371/journal.pntd.0009888)
Supplement: S3 Table — (DOCX) [file pntd.0009888.s004.docx]

## S3 Table. Coefficient estimates for the negative binomial mixed effects model fitted to *Trichuris trichiura* fecal egg counts. Estimates derived from fitting the Bayesian model to fecal egg count data on *Trichuris trichiura* collected before and after administration of a single 400 mg oral dose of albendazole in three study sites, Ethiopia, Lao PDR and Pemba Island (Tanzania).

| **Variable** | **Posterior mean (95% CIr^a^)** | $\hat{R}$ | |
| --- | --- | --- | --- |
| Intercept | 0.68 (-0.41, 1.64) | 1.00 | |
| Country |  |  | |
| Ethiopia | 0^b^ | NA | |
| Lao PDR | 0.70 (-0.42, 1.98) | 1.00 | |
| Pemba Island | 3.21 (2.15, 4.45) | 1.00 | |
| Sex |  |  | |
| Female | 0 | NA | |
| Male | -0.06 (-0.31, 0.19) | 1.00 | |
| Age |  |  | |
| 6-9 years | 0 | NA | |
| 10-12 years | 0.09 (-0.24, 0.40) | 1.00 | |
| 13-14 years | -0.11 (-0.52, 0.30) | 1.00 | |
| Coinfection |  |  | |
| Single infection | 0 | NA | |
| *Ascaris lumbricoides* | 0.14 (-0.17, 0.46) | 1.00 | |
| hookworm | 0.35 (0.06, 0.66) | 1.00 | |
| Treatment |  |  | |
| Baseline | 0 | NA | |
| Follow-up | -1.08 (-1.53, -0.62) | 1.00 | |
| Country*Treatment |  |  | |
| Ethiopia | 0 | 0 | |
| Lao PDR | 0.24 (-0.24, 0.72) | 1.00 | |
| Pemba Island | 1.27 (0.90, 1.64) | 1.00 | |
| Sex*Treatment |  |  |  |
| Female | 0 | NA |  |
| Male | 0.10 (-0.16, 0.36) | 1.00 |  |
| Age*Treatment |  |  |  |
| 6-9 years | 0 | NA |  |
| 10-12 years | -0.29 (-0.60, 0.01) | 1.00 |  |
| 13-14 years | -0.12 (-0.56, 0.32) | 1.00 |  |
| Follow up*Treatment |  |  |  |
| > 2 weeks | 0 | NA |  |
| 1-2 weeks | 0.00 (-0.29, 0.30) | 1.00 |  |
| Coinfection*Treatment |  |  |  |
| Single infection | 0 | NA |  |
| *Ascaris lumbricoides* | -0.22 (-0.54, 0.10) | 1.00 |  |
| hookworm | 0.01 (-0.29, 0.30) | 1.00 |  |
| *Random effects hyperparameters* |  |  |  |
| SD^d^ individual intercept | 1.29 (1.20, 1.40) | 1.00 |  |
| SD individual treatment response | 1.26 (1.15, 1.37) | 1.00 |  |
| Correlation intercept & treatment response | -0.18 (-0.30, -0.07) | 1.00 |  |
| SD school intercept | 0.56 (0.20, 1.25) | 1.00 |  |
| Overdispersion parameter | 16.93 (14.49, 19.71) | 1.00 |  |

^a^ credible interval; ^b^ coefficient for reference category set to 0; ^c^ not applicable; ^d^ standard deviation.
