# Supplementary material for: Individual responses to a single oral dose of albendazole indicate reduced efficacy against soil-transmitted helminths in an area with high drug pressure
Source: PLoS Negl Trop Dis. 2021 Oct 19;15(10):e0009888. doi: 10.1371/journal.pntd.0009888 (PMC8555840; doi:10.1371/journal.pntd.0009888)
Supplement: S4 Table — (DOCX) [file pntd.0009888.s005.docx]

## S4 Table. Coefficient estimates for the negative binomial mixed effects model fitted to hookworm fecal egg counts. Estimates derived by fitting the Bayesian model to fecal egg count data on hookworm collected before and after administration of a single 400 mg oral dose of albendazole in three study sites, Ethiopia, Lao PDR and Pemba Island (Tanzania).

| **Variable** | **Posterior mean (95% CrI^a^)** | $\hat{R}$ |
| --- | --- | --- |
| Intercept | 0.84 (-0.12, 1.96) | 1.00 |
| Country |  |  |
| Ethiopia | 0^b^ | NA |
| Lao PDR | 1.54 (0.35, 2.57) | 1.00 |
| Pemba Island | -0.47 (-1.58, 0.66) | 1.01 |
| Sex |  |  |
| Female | 0 | NA |
| Male | 0.34 (-0.03, 0.70) | 1.00 |
| Age |  |  |
| 6-9 years | 0 | NA |
| 10-12 years | 0.55 (0.04, 1.05) | 1.01 |
| 13-14 years | 0.89 (0.35, 1.47) | 1.00 |
| Coinfection |  |  |
| Single infection | 0 | NA |
| *Ascaris lumbricoides* | -0.60 (-1.00, -0.19) | 1.00 |
| *Trichuris trichiura* | 0.32 (-0.15, 0.78) | 1.01 |
| Treatment |  |  |
| Baseline | 0 | NA |
| Follow-up | -6.55 (-8.52, -4.76) | 1.01 |
| Country*Treatment |  |  |
| Ethiopia | 0 | NA |
| Lao PDR | 0.18 (-1.19, 1.64) | 1.00 |
| Pemba Island | 2.07 (0.61, 3.55) | 1.00 |
| Sex*Treatment |  |  |
| Female | 0 | NA |
| Male | 0.82 (-0.07, 1.71) | 1.01 |
| Age*Treatment |  |  |
| 6-9 years | 0 | NA |
| 10-12 years | -0.64 (-1.82, 0.57) | 1.00 |
| 13-14 years | -0.14 (-1.40, 1.20) | 1.00 |
| Follow up*Treatment |  |  |
| > 2 weeks | 0 | NA |
| 1-2 weeks | -0.56 (-1.48, 0.32) | 1.00 |
| Coinfection*Treatment |  |  |
| Single infection | 0 | NA |
| *Ascaris lumbricoides* | 0.12 (-0.86, 1.09) | 1.01 |
| *Trichuris trichiura* | 0.63 (-0.43, 1.66) | 1.00 |
| *Random effects hyperparameters* |  |  |
| SD^d^ individual intercept | 1.87 (1.72, 2.02) | 1.01 |
| SD individual treatment response | 3.45 (2.97, 4.04) | 1.00 |
| Correlation intercept & treatment response | -0.15 (-0.30, 0.01) | 1.00 |
| SD school intercept | 0.43 (0.03, 1.22) | 1.01 |
| Overdispersion parameter | 16.32 (12.66, 20.70) | 1.00 |

^a^ credible interval; ^b^ coefficient for reference category set to 0; ^c^ not applicable; ^d^ standard deviation
