# Supplementary material for: Individual responses to a single oral dose of albendazole indicate reduced efficacy against soil-transmitted helminths in an area with high drug pressure
Source: PLoS Negl Trop Dis. 2021 Oct 19;15(10):e0009888. doi: 10.1371/journal.pntd.0009888 (PMC8555840; doi:10.1371/journal.pntd.0009888)
Supplement: Attachment — Submitted filename: Walker et al 2021 R1 Response to reviewers.docx [file pntd.0009888.s006.docx]

**Reviewer #1: PNTD-D-21-00696**

**An interesting paper combining population- and individual patient-level assessment of responses to albendazole by different STHs.**

We thank the Reviewer for these appreciative comments.

**-   What does “publicly available individual patient data” mean? Where are these datasets and can they be accessed by others? Refs 18, 19, 20 do not direct to raw data sources. Are these data in the IDDO data platform? If so it should be explicitly stated from the outset.**

The data can be accessed at <https://doi.org/10.1371/journal.pntd.0007471.s004>, which is part of the supplementary information of the publication by Vlaminck et al, 2019. The data have also been submitted to the IDDO data platform and are currently being curated.

**-   Confused about the numbers. “We re-analyzed 457 individual patient data” in the Abstract does not correspond to any of the numbers reported later in the paper. Lines 144-145: “available for 645 children across the three study sites (Ethiopia: 161 cases; Lao PDR: 239 cases; Pemba Island: 245 cases” – see also next question.**

We thank the Reviewer for this picking up this typographical error. The 645 refers to the number of individuals who tested positive for *any* STH by at least one of the four diagnostic methods used in the study; we have amended the Abstract to reflect this. We have also checked, and where necessary, clarified the numbers reported throughout the text. We have also included information in Table 1 on the number of individuals positive by any diagnostic and positive by Kato-Katz.

**-   Did this analysis include all the patients from those 3 trials? From the text and Table 1 I get the following numbers of infections. It would also be useful to have both number of infections and number of patients in the same table. Coinfections occurred. Are these a total of 1354 infections in 645 (line 145) or 441 (line 149) or 457 (Abstract) children?
    	Ethiopia  Lao     Pemba   total/species
    A. lumbricoides  137     111     193     441
    T. trichiura        106     105     245     456
    Hookworms   90      228     139     457
    total/country       333     444     577     1354**

We thank the Reviewer for this question. The total number of individuals positive for any species (by any diagnostic) was 645. The numbers in the table (441, 456 and 457) indicate the number of patients from the three trials who were positive for each species by any diagnostic. The analysis in this paper (and in the preceding paper by Vlaminck et al., 2019) was species-specific, and therefore the data from 441, 456 and 457 individuals were used for the analysis of *A. lumbricoides*, *T. trichiura* and hookworm respectively. This is identical to the approach used in Vlaminck et al., 2019. To further clarify, we have updated Table 1 to include the additional information requested.

**-   Is it correct to assume “duplicate KK” is performed on a single stool sample?**

Yes, this is correct. We have made this is clearer in the revised manuscript.

**-   While understanding the rational for analyses being based on duplicate KK, was any sensitivity analysis done using another technique? This is relevant since they found “a significant correlation between the individual response and the infection intensity prior to drug administration (the individual response decreasing as a function of increasing infection intensity)”, while at the same time “egg counts that were significantly different from those reported by Kato-Katz thick smear (Mini-FLOTAC and FECPAKG2)”.**

The comparison of the different diagnostics for estimating the population-based ERR is described and discussed in detail by Vlaminck et al., 2019. In the present study, we focused on KK only for the reasons outlined in the text. Namely, that: “(i) Kato-Katz thick smear is the most commonly applied method for monitoring and evaluation activities in STH MDA programs; (ii) of all the applied methods in these trials, Kato-Katz thick smear is the only method that has been recommended so far by the World Health Organization (WHO) to assess therapeutic efficacy…”

**-   As hookworm infections were found to be caused by N. americanus, it may be useful to discuss different susceptibilities of N. americanus vs A. duodenalis.**

This issue was discussed in the original paper by Vlaminck et al., 2019, namely, that there was a slightly higher cure rate against *Ancylostoma* compared to *Necator* infections, albeit based on only 8 children with mixed infections. We feel that revisiting this issue—based on such a small sample size—is out of scope of the current manuscript. We have edited the manuscript to point the interested reader towards the Vlaminck et al., 2019 paper, “…see Vlaminck et al. [19] for discussion on the potential differential susceptibility of *N. americanus* and *Ancylostoma* species to ALB”.

**-   Re: ERR. It’d be useful to have “The 95% confidence intervals (CIs) were calculated using a non-parametric block bootstrap approach [26]” in the M&Ms and not (just) in the Table 1 legend.**

We thank the Reviewer for this suggestion. We have now moved this description into the Methods section.

**-   Re: WHO response classification criteria.**

**o   While it is customary to use ERR, whether this or rather 95%CIs should be used is still an unresolved issue.**

We agree with the Reviewer that this is indeed an unresolved issue. In the manuscript the classification was made on the point estimate only. As a standard practice we also reported the 95CIs.

**o   It is questionable to apply WHO classification criteria developed for population-level response for individual response distribution**

In absence of any alternative, we feel that applying the WHO classification criteria is justified. Nevertheless, we have made it clear in the revised manuscript that these criteria were developed for population-based ERRs.

**o   It is also questionable to keep using bespoke efficacy criteria that have been developed out of conveniency for poorly-effective drugs (notably: 50% ERR for ALB on T. trichiura) and continue to pretend we are happy with that.**

In absence of any alternative that is supported by the wider community, we feel that use of these criteria is justified. We are however sympathetic to the Reviewer’s assertion on the poor efficacy of ALB against *T. trichiura* and, as we conclude strongly in the manuscript, global elimination goals for STH will not be met in areas where *T. trichiura* is highly endemic using the current single drug paradigm.

**-   I am very happy to see individual-level response finally being adopted to assess the level of efficacy of anthelminthics.**

We thank the Reviewer for this comment, which we are very pleased to read. **Reviewer #2: This a very interesting and beautiful secondary application of Bayesian count models to estimate effect of patient’s and study characteristics on treatment efficacy on egg reduction rates from 3 pooled study data. The text is well writtten despite the complexity of the subject.**

We thank the Reviewer for these supportive remarks.

**There are some minor comments to slightly improve the manuscript.
    Page 9, FEC: the authors should mention how FEC is obtained as it is stated later at several occasion especially in the legend of figure 1, first sentence after figure 1 and legend of table 2**

We have clarified the definition of fecal egg count as being “the number of eggs counted from a single Kato-Katz slide, such that two FECs are measured from duplicate Kato-Katz slides from each individual at each time point.”

**Page 12, table 1 legend: the authors define the number of cases as the number of participants identified with any four diagnostics. However, they have previously stated in the methods that the ERR is calculated only with the KK evaluation. Therefore, they should explain how the ERR is calculated in the absence of KK evaluation and with one of the 3 other diagnostic tool.**

We calculated ERRs using only FEC data from KK. The comparison of the different diagnostics for estimating the population-based ERR is described and discussed in detail by Vlaminck et al., 2019. We have clarified in the legend of Table 1 how the calculation was made, while also indicating how many individuals were positive by KK or any diagnostic and also how many were screened for infection.

**Reviewer #2: In the PLOS NTD figure legend tips it is stated to “avoid lenghty description of methods”. However figure and table legends describe elements of methodology not described in the methods paragraph. This could be improved.**

We have now moved the description of the bootstrap methodology used for calculating the 95CIs from the Table 1 legend to the Methods section. In addition, we have further simplified methodological descriptions across all legends.

**Reviewer #3: 1.     Not clear what the authors are referring to with, “..dose-response relationships against the different STH infections” (page 6, line 63).**

We are referring to the impact of increasing the dose on therapeutic efficacy. This has now been made clearer in the revised manuscript, “…impact of dose on the drug response for the different STH infections”.

**2.  The manuscript needs to be reviewed closely for typos, etc**

We have thoroughly revised the manuscript for typographical errors.
